# Frequency of Smoking and Marginal Bone Loss around Dental Implants: A Retrospective Matched-Control Study

**DOI:** 10.3390/jcm12041386

**Published:** 2023-02-09

**Authors:** Amir Ali, Ammar Al Attar, Bruno Ramos Chrcanovic

**Affiliations:** 1 Faculty of Odontology, Malmö University, 214 21 Malmö, Sweden; 2 Department of Prosthodontics, Faculty of Odontology, Malmö University, 214 21 Malmö, Sweden

**Keywords:** smoking, smoking frequency, dental implants, marginal bone loss, matched-control, retrospective study

## Abstract

This dental record-based retrospective study aimed to compare the marginal bone loss (MBL) around dental implants in a group of smokers in relation to a matched group of non-smokers, with a special focus on five different frequencies of daily smoking (non-smokers, and frequency of 1–5, 6–10, 11–15, and 20 cig./day). Only implants with a minimum of 36 months of radiological follow-up were considered. Univariate linear regression models were used to compare MBL over time between 12 clinical covariates, after which a linear mixed-effects model was built. After matching of the patients, the study included 340 implants in 104 smokers, and 337 implants in 100 non-smokers. The results suggested that smoking degree (greater MBL for higher degrees of smoking), bruxism (greater MBL for bruxers), jaw (greater MBL in maxilla), prosthesis fixation (greater MBL for screw-retained prosthesis), and implant diameter (greater MBL for 3.75–4.10 mm) had a significant influence on MBL over time. There appears to be a positive correlation between the degree of smoking and the degree of MBL, meaning, the higher the degree of smoking, the greater the MBL. However, the difference is not apparent for different degrees of smoking when this is high, namely above 10 cigarettes per day.

## 1. Introduction

The estimated number of smokers in the world at the year of 2019 was 1.1 billion people. Since 1990, the prevalence of smokers has fallen because of the growth population, but the number of smokers has increased. Smoking is a harmful habit and causes around 8 million deaths each year [1].

In addition to many serious illnesses, such as cancer, stroke, cardiovascular diseases, and other health problems, smoking cigarettes has a huge impact on oral health [2]. In addition to raising the risk of periodontal disease, smoking cigarettes is linked to poor oral health [3]. Cigarette smoking has an impact on increased tooth mobility [4] and there is a greater risk of tooth loss among smokers than among non-smokers [5,6]. Smoking has also been associated with a higher risk of dental implant failure and a worse effect on the level of marginal bone around implants in comparison to non-smokers [7,8].

A relationship seems to exist between frequency of smoking and periodontal disease. Heavy smoking was found to have high prevalence and severity of periodontitis as compared with moderate and light smokers [9].

When it comes to dental implants, studies have compared marginal bone loss (MBL) between groups of smokers and non-smokers concerning some further sub-groups, such as turned vs. oxidized implants [10], periodontally healthy vs. periodontally compromised patients [11], different prosthetic configurations of implant-supported overdentures in the mandible [12], diabetic vs. non-diabetic patients [13], and immediate and delayed loading [14]. For the great majority of the cases, smoking resulted in greater MBL than in non-smokers, regardless of these aforementioned sub-divisions. A recent review on the effect of smoking on dental implants included 292 studies, of which 32 provided information comparing the mean MBL between smokers and non-smokers [8]. Taking together the results of these 32 studies, it was observed that implants placed in smokers presented a statistically significant mean 0.580 mm higher MBL than the implants placed in non-smokers. As each and every one of these 32 studies provided clear information on MBL between these two groups, a simple dichotomous analysis on MBL between smokers and non-smokers would not present any novelty. It is not clear, however, how the varying frequency of daily smoking would affect marginal bone loss (MBL) around dental implants.

Therefore, the aim of the present retrospective study was to compare the MBL around dental implants in a group of smoker patients in relation to a matched group of non-smokers, taking into consideration several variables, with a special focus on different frequencies of daily smoking. Patients in this study were classified into five groups according to the different frequencies of daily smoking.

## 2. Materials and Methods

### 2.1. Research Question

The focused question was elaborated by using the PICO format (Participants, Interventions, Comparisons, Outcomes): “Do smokers undergoing implant-prosthetic rehabilitation present a higher MBL in comparison to non-smokers?”

The null hypothesis was that there would be no difference in MBL between smokers, different frequencies of smoking, and non-smokers, against the alternative hypothesis of a difference.

### 2.2. Materials

This retrospective study included patients treated with dental implants during the period 1980–2018 at one specialist clinic (Clinic for Prosthodontics, Centre of Dental Specialist Care, Malmö, Sweden). This study was based on data collection from patients’ dental records. The implants were placed by specialist dentists in oral surgery, and dentists performing the prosthetic treatment were specialists in prosthodontics.

The study was approved by the regional Ethical Committee, Lund, Sweden (Dnr 2014/598; Dnr 2015/72). The present retrospective study followed the STROBE guidelines for observational studies and was registered at https://clinicaltrials.gov under the registration number NCT02369562, last updated on 3 May 2019.

### 2.3. Definitions

For this study, patients smoking a minimum of one cigarette per day (an everyday smoker [15]) were classified as smokers, established at the clinical appointment of the patient when the anamnesis was performed.

MBL was defined as loss, in an apical direction, of alveolar bone marginally adjacent to the dental implant, in relation to the marginal bone level initially detected after the implant was surgically placed [16].

The diagnosis of bruxism was established in a previous study [17], in which the patients of the aforementioned database suspected to be bruxers were called back for one clinical appointment to get the minimum information in order to diagnose the patients as ‘probable bruxers’ (self-report/anamnesis + clinical examination).

As the standard protocol in the clinic, the patients’ dental hygiene was followed up by a dental hygienist within 6 months after the final implant-supported/retained restoration. Each patient then attended a dental hygiene recall program based on individual needs.

### 2.4. Inclusion and Exclusion Criteria

Only implants that did not present loss of osseointegration and with baseline radiographs taken within 12 months after implant placement and with a minimum of 36 months of radiological follow-up were considered for the analysis of MBL. Negative values of MBL correspond to bone loss.

Patients with all modern types of threaded implants with cylindrical or conical design were included. Zygomatic implants were not included in the study, nor were implants detected in radiographies but lacking basic information about them in the patients’ files.

Patients were excluded if they had a history of periodontitis and/or were treated for periodontal disease. It is important to take note that as standard, all patients receiving implants at the Specialist Clinic for Prosthodontics were periodontally healthy at the time of implant installation. Patients with either a history or signs of periodontal disease were treated at the Specialist Clinic for Periodontology, where they later could or not receive dental implants, according to individual needs/indications. These patients were not included in the present study.

### 2.5. Data Collection

The data were directly entered into a SPSS file (SPSS software, version 28, SPSS Inc., Chicago, IL, USA) as the dental records of the patients were being read, and it consisted of the following variables: patient age at implant installation, patient’s sex, probable bruxism (yes/no), diabetes status (absent, type 1, type 2), smoking habit (yes/no), number of cigarettes/day (frequency of 0 [non-smoker], 1–5, 6–10, 11–15, and 20 cigarettes per day), implant location (regarding jaw [maxilla/mandible, anterior/posterior—anterior region comprised incisors and canines] and tooth region [incisor, canine, premolar, molar]), implant diameter (three groups: 3.00–3.50, 3.75–4.10, and 4.30–5.00 mm), implant surface (turned/machined, modified), prosthesis type (single crown, fixed dental prosthesis (FDP) of 2 to 6 prosthetic units, FDP of 7 to 10 units, full-arch, overdenture), prosthesis fixation (cemented, screwed), and follow-up time.

### 2.6. Formation of a Matched Group

Since the division of all initial patients into groups would generate extremely unbalanced groups and the variance was not homogenous between them, the two groups were therefore not expected to be comparable with respect to important covariates [18], and then methods were used to match patients and implants between smokers to non-smokers patients. Matching minimizes the chance that any differences between the study and the control groups are a result of differences on the matching variables, thus reducing selection bias.

The matching was performed using the ‘case control matching’ function in SPSS, and the matches were selected based on similarities in (a) patient’s age at the time of the surgery, (b) number of implants, and (c) total radiological follow-up time. As there were no perfect matches in a first matching attempt considering all three variables, some tolerance was set for the predictors: ±5 years for the patient’s age, ±2 implants, and ±12 months for the total radiological follow-up time. Thus, a little variance of these predictors between the groups was expected.

### 2.7. Marginal Bone Level Evaluation

The evaluation of the variation of the marginal bone level over time was performed according to a previous study [19]. Reproducible intra-oral radiographs were used. When there were no available digital radiographies from the baseline appointment, the analogue periapical radiographies were scanned at 1200 dpi (Epson Perfection V800 Photo Color Scanner; Nagano, Japan). Marginal bone level (MBL) was measured after calibration based on the inter-thread distance of the implants. Information about the inter-thread distance was obtained from the implant catalogue of each implant manufacturer. Measurements were taken from the implant-abutment junction to the marginal bone level, at both mesial and distal sides of each implant, and then the mean value of these two measurements was considered. MBL was calculated by comparing bone-to-implant contact levels to the radiographic baseline examination. The Image J software (National Institute of Health, Bethesda, MD, USA) was used for all measurements.

The sets of radiographs for every patient were codified, and the authors who performed the radiological measurements (A.A., A.A.A.) were blinded to the smoking habit for every patient.

### 2.8. Calibration

An initial calibration concerning MBL was performed between the authors. The process was done for 10 random samples from the cohort group and verified after the measurement of each sample. At the end of the process, the measurements from the different individuals were considered approximate enough to each other, with agreement between examiners set at >80% of the distance in millimeters.

### 2.9. Sample Size Calculation

The calculation of the sample size was based on the results of a study [20], which observed a mean MBL ± SD for smokers of 0.57 ± 0.93 mm, and 0.30 ± 0.58 mm for non-smokers. There was a need for 186 implants in each group having set alpha (α) at 0.05 and power at 80%. The sample size calculation was performed with ClinCalc.com.

### 2.10. Statistical Analyses

The mean, standard deviation, and percentages were presented as descriptive statistics. The Kolmogorov–Smirnov test was performed to evaluate the normal distribution of the variables, and Levene’s test evaluated homoscedasticity. The performed tests for two independent groups were Student’s *t*-test or the Mann–Whitney test, and paired-samples *t*-test or Wilcoxon signed-rank test for two dependent groups, depending on the normality. Pearson’s chi-squared test or Fisher’s exact test was used in the analysis of contingency tables of categorical data of independent groups, and McNemar’s test for dependent groups.

Univariate linear regression models were used to compare MBL over time between clinical covariates. The estimation of MBL over time (dependent variable) was expressed in a single linear regression equation for each of the categories of each independent variable (smoking, diabetes, bruxism, sex, age, jaw, jaw region, tooth region, implant diameter, implant surface, prosthesis type, prosthesis fixation). For the present study, the linear regression equation was expressed as *y* = *b* + *ax*, where ‘*y*’ is the estimated MBL over time, ‘*b*’ is the estimated intercept at the *y*-axle in the scatter plot, ‘*a*’ is the estimated MBL per each one month of follow-up, and ‘*x*’ is the number of months of follow-up. Thus, if one would like to estimate the MBL of a certain category of a certain variable at, for example, 100 months of follow-up, ‘*x*’ is replaced by the value of 100 in the equation given for that particular category and variable.

In order to verify multicollinearity, a correlation matrix of all of the predictor variables was scanned, to see whether there were some high correlations among the predictors. Collinearity statistics obtaining variance inflation factor (VIF) and tolerance statistic were also performed to detect more subtle forms of multicollinearity. A linear mixed-effects model was built with all variables that were moderately associated (*p* < 0.10) with MBL in the univariate linear regression models. A mixed-effects model was used in order to take into consideration that some patients had more than one implant-supported prosthesis, as multiple observations within an individual are not independent of each other. Multiple testing corrections for *p*-values were performed by the Bonferroni adjustment.

The degree of statistical significance was considered *p* < 0.05. Data were statistically analyzed using the Statistical Package for the Social Sciences (SPSS) version 28 software (SPSS Inc., Chicago, IL, USA).

## 3. Results

The cohort group initially included 760 implants installed in 210 patients, 105 smokers and 105 non-smokers. The periapical radiographs for one smoker and five non-smoker patients were excluded for not being of sufficient quality. Therefore, 204 patients were included in the present study (104 smokers, 100 non-smokers), with 677 dental implants (340 in smokers, 337 in non-smokers). Most of the implants of the study were Brånemark MK implants (Nobel Biocare, Göteborg, Sweden), totaling 590 implants (382 turned/machined and 208 TiUnite implants).

The mean age (±SD) of the 204 patients was 54.6 ± 14.7 years (min–max, 15.2–81.3) at the day of the implant surgical placement. The patients were followed up clinically for a mean (±SD) of 162.4 ± 75.6 months (min–max, 40.4–364.2), and radiographically for a mean (±SD) of 133.1 ± 75.0 months (min–max, 39.6–364.2).

Table 1 shows the descriptive data of the implants included in the study. The variable patient’s age was divided into three categories each, based on the 33.3 and 66.7 percentiles of sample distribution, in order to generate groups of more balanced sample sizes. There was a difference in distribution of implants of different surfaces between the groups, as well as for the age groups of the patients, and the distribution of patients with different types of diabetes. The site of 20 implants were submitted to augmentation procedure simultaneous to implant placement, and 12 implant sites were submitted to autogenous graft varying from 4.7 to 10 months previous to the implant placement surgery.

The total number of marginal bone level double measurements (mesial + distal sides of each implant) was 4324. Table 2 shows data on MBL distributed by different periods of follow-up, separated by different frequencies of daily smoking. The general picture was an increase in MBL with time of follow-up and with the frequency of smoking. It is important to stress that not all implants in all patients were followed up for the same period of time.

**Figure 1:**
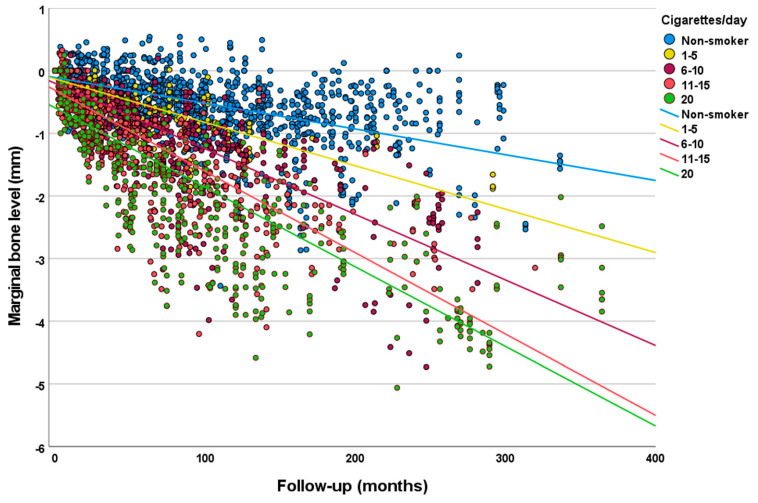
Scatter plot comparing the marginal bone level over time between implants placed in patients with different degrees of smoking (linear regression).

**Figure 2:**
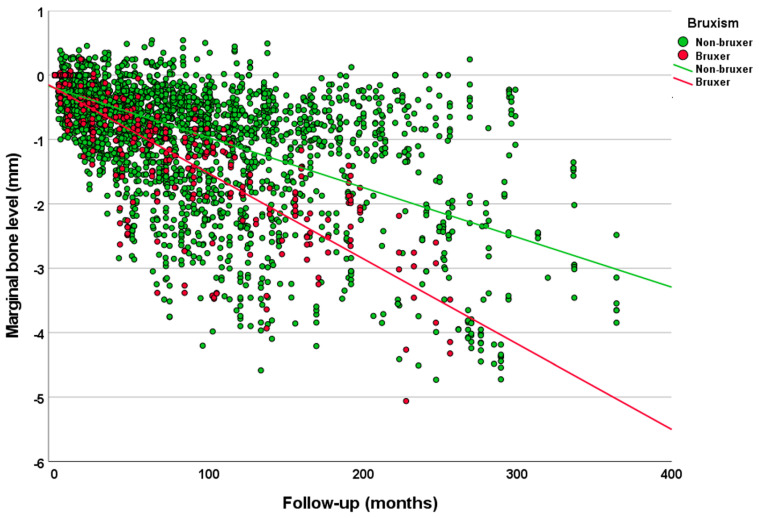
Scatter plot comparing the marginal bone level over time between implants placed in bruxers and non-bruxer patients (linear regression).

Analysis of the univariate linear regression analysis (Table 3) showed that the estimated MBL over time was statistically significantly different between the categories of the following variables: smoking degree (greater MBL for higher degrees of smoking) (Figure 1); bruxism (greater MBL for bruxers) (Figure 2); patient’s sex (greater MBL in men); patient’s age at the time of implant placement (greater MBL for the range 51–61 years); jaw (greater MBL in maxilla); jaw region (greater MBL in posterior region); implant diameter (greater MBL for regular diameter implants); implant surface (greater MBL for implants with modified surface); prosthesis type (greater MBL for FDP 2–6 units and full-arch fixed prosthesis); and prosthesis fixation (greater MBL for screw-retained prosthesis). Most the categories had a moderate degree of linear correlation (R^2^ linear) with MBL over time. Diabetes and tooth region were not statistically significant.

The results of the linear mixed-effects model (Table 4) suggested that smoking degree (greater MBL for higher degrees of smoking), bruxism (greater MBL for bruxers), jaw (greater MBL in maxilla), implant diameter (greater MBL for regular diameter implants), and prosthesis fixation (greater MBL for screw-retained prosthesis) had a statistically significant influence on MBL over time.

## 4. Discussion

The aim of the present retrospective study was to investigate the influence of smoking on MBL at dental implants. According to the results of the present study, there was a statistically significant difference in MBL between smokers and non-smokers. Therefore, the null hypothesis was rejected. Some factors may explain these results.

Much is believed to be associated with the negative effects of the smoking toxins on bone metabolism and osteogenesis, and on angiogenesis. Cigarette smoke exposure results in the reduction of the bone trabeculae thickness which is associated with a decrease in mineralizing surface as well as in the mineral deposition rate [21]. Cigarette smoke also disturbs angiogenesis by inhibiting several biochemical and physiological processes that results in abnormal blood supply to tissues, which in turn decreases repair of damaged tissues and remodeling [22]. There is an increased risk of peri-implantitis in smokers compared to non-smokers [23]. Systematic reviews with meta-analysis on the subject including 107 and 292 studies, respectively, suggested that smoking has a worse effect on the level of marginal bone around implants in comparison to non-smokers [7,8].

The results of the present study suggest that there appears to be a positive correlation between the degree of smoking and the degree of MBL. A study suggested that smoking lowers bone mineral density and is a result of decreased calcium absorption associated with secondary hyperparathyroidism and increased bone resorption. Moreover, a significant increase in bone remodeling markers was observed in heavy smokers in comparison to light smokers, suggesting that the negative effect of smoking on bone may be higher with an increase in smoking frequency [24]. The results of a study showed that individuals smoking up to 10 cigarettes a day presented similar MBL to non-smokers [25]. However, this study had mean follow-up of less than four years and investigated a very limited number of implants, with a considerable unbalance between the number of implants in smokers versus non-smokers. Many studies that investigated the possible effect of smoking on MBL around implants failed to report the level of smoking of the included smoker individuals [8]. Even when this information is provided, there is a lack of homogeneity of what would be considered a ‘light smoker’ and a ‘heavy smoker’. The positive correlation between the degree of smoking and the degree of MBL in the present study was more clearly observed up to a certain limit, and patients who used to smoke between 11 and 20 cigarettes per day showed similar estimated MBL over time. This might suggest that the deleterious effects of smoking on MBL may not significantly increase after an already high degree of smoking. However, more studies are necessary in order to verify whether the negative effect of smoking on the marginal bone levels may tend to level above a certain degree of cigarette smoking.

The impact of smoking on MBL may also be associated with the duration of smoking, although this was not investigated in the present study. Cigarette smoking contributes to the constriction and damage of arteries, and promotes endothelial dysfunction and by altering lipoprotein metabolism, coagulation, and platelet function [26]. Some studies showed that the effect of cumulative smoking exposure on arterial damage has a dose–response relationship [27,28]. It is likely that the nocive effects of smoking are cumulative also on bone [29]. The cumulative negative effect of pack-years of smoking might have a significant negative effect on MBL as well, as the long-term maintenance of normal bone metabolism and vascular health may play an important role in the maintenance of bone around dental implants.

The greater MBL in bruxers in relation to non-bruxers could be associated with the absence of a periodontal ligament around dental implants, limiting the amount of feedback the central nervous system receives, causing a reduction in the tactile sensitivity around implants [30]. Therefore implant-supported restorations are more likely to be subjected to higher loads during bruxism because of this reduced tactile sensitivity around implants [31,32,33,34], which could in theory negatively affect the marginal bone level. The results of the first clinical study comparing MBL in a group of bruxers in relation to a matched group of non-bruxers suggested that bruxism increases the risk of MBL over time [19]. The fact that a patient had an occlusal splint was not a reliable piece of information, as confirmed in the study that previously investigated the bruxers in this cohort [17]. There were cases where patients had an occlusal splint but were used only eventually, or used only during the first two weeks, or never used it.

There was an estimated greater MBL over time around implants in the maxilla in comparison to implants in the mandible. This could be related to the differences in bone quality between the jaws [35]. Smoking may be a significant risk factor with adverse effects on implants in areas of loose trabecular bone but may not be as significant for dense bone sites, such as the mandible [36].

Another finding was the greater estimated MBL over time for regular diameter implants in comparison to narrow and wide diameter implants. This goes against the results of a study that observed greater MBL with larger diameter implants [37] and could be related to the extremely unbalanced groups of implants of different diameter ranges. The authors of this previous study [37] hypothesized that the posterior regions of the jaw more often present an alveolar ridge wide enough to receive wide implants, in a region where tissue architecture is different and mechanical loads are higher. According to them, larger implants are therefore expected to be subjected to higher compressive forces, and these may have caused more resorption.

The last factor to show a statistically significant difference in the estimated MBL over time was the prosthesis fixation type, with screw-retained, implant-supported restoration showing greater MBL than around implants supporting cemented restorations. In vitro and in vivo studies have suggested that there is minimal stress exertion on the implant and the crestal bone around it with cement-retained prostheses in comparison to screw-retained prostheses [38,39,40]. However, we cannot draw any conclusions from this, since a disproportionate amount of screw-retained and cement-retained implant-supported prostheses were used for this retrospective study. There should be a study dedicated to this subject that accounts for both types of anchoring prostheses to the implant.

As limitations of the present study, this was a dental record-based retrospective study. The nature of a retrospective study inherently results in flaws. These problems were manifested by the gaps in information and incomplete records. Furthermore, all data rely on the accuracy of the original examination and documentation. Items may have been excluded in the initial examination or not recorded in the dental chart. Moreover, the smoking habit was assessed at the time of implant installation and might have changed over time for each patient.

## 5. Conclusions

Smoking negatively affect the MBL at implants. There appears to be a positive correlation between the degree of smoking and the degree of MBL, meaning, the higher the degree of smoking, the greater the MBL. However, this correlation is more clearly observed up to a certain limit. The difference is not apparent for different degrees of smoking when this is high, namely above 10 cigarettes per day.

Other factors that are suggested to negatively affect MBL are bruxism, implant location (in the maxilla), implant diameter (greater MBL for 3.75 mm–4.10 mm), and screwed implant-supported prostheses.

## Figures and Tables

**Table 1:** Descriptive data of the implants included in the study, separated by group. The statistical unit is the implant, not the patient.

Factor	Smokers, Implants (%)	Non-Smokers, Implants (%)	*p* Value
Follow-up ^a^ (months)			
(mean ± SD)	128.8 ± 77.1	139.4 ± 72.4	0.056 ^b^
Age			
(mean ± SD)	53.0 ± 14.2	56.3 ± 15.1	0.063 ^b^
Sex			
Male	151 (44.8)	168 (49.4)	0.230 ^c^
Female	186 (55.2)	172 (50.6)	
Age (years)			
<50	125 (37.1)	100 (29.4)	0.023 ^c^
51–61	112 (33.2)	107 (31.5)	
>62	100 (29.7)	133 (39.1)	
Jaw			
Maxilla	187 (55.5)	182 (53.5)	0.609 ^c^
Mandible	150 (44.5)	158 (46.5)	
Jaw region			
Anterior	191 (56.7)	204 (60.0)	0.380 ^c^
Posterior	146 (43.3)	136 (40.0)	
Tooth region			
Incisor	132 (39.2)	128 (37.6)	0.190 ^c^
Canine	60 (17.8)	76 (22.4)	
Premolar	120 (35.6)	102 (30.0)	
Molar	25 (7.4)	34 (10.0)	
Implant diameter			
3.00–3.50 mm	30 (8.9)	25 (7.3)	0.441 ^c^
3.75–4.10 mm	301 (89.3)	312 (91.8)	
4.30–5.00 mm	6 (1.8)	3 (0.9)	
Implant surface			
Turned	154 (45.7)	228 (67.1)	<0.001 ^c^
Modified	183 (54.3)	112 (32.9)	
Prosthesis type ^b^			
Single crown	44 (13.1)	44 (12.9)	0.181 ^c^
FDP 2–6 units	115 (34.1)	102 (30.0)	
FDP 7–10 units	10 (3.0)	16 (4.7)	
Full-arch	168 (49.8)	174 (51.2)	
Overdenture	0 (0)	4 (1.2)	
Prosthesis fixation ^b^			
Cemented	31 (9.3)	37 (11.3)	0.412 ^c^
Screwed	301 (90.7)	291 (88.7)	
Bruxism ^d^			
No	292 (89.8)	312 (93.4)	0.098 ^c^
Yes	33 (10.2)	22 (6.6)	
Diabetes ^d^			
No	288 (88.1)	309 (91.2)	<0.001 ^c^
Type 1	0 (0)	13 (3.8)	
Type 2	39 (11.9)	17 (5.0)	

^a^ Radiological follow-up; ^b^ Wilcoxon signed-rank test; ^c^ comparison of the distribution of implants, among the categories of each factor, between smokers and non-smokers; ^d^ for the cases with available information. SD—standard deviation. FDP—fixed dental prosthesis.

**Table 2:** Data on marginal bone loss distributed by different periods of follow-up, separated by different frequencies of daily smoking. Values in millimeters. Negative values correspond to bone loss.

Follow-Up (Years)	Non-Smoker	1–5 Cig/Day	6–10 Cig/Day	11–15 Cig/Day	20 Cig/Day
		mean ± SD (min, max)			
0–1	−0.02 ± 0.11 (−0.97, −0.02)	−0.07 ± 0.15 (−0.83, 0.11)	−0.10 ± 0.17 (−0.80, 0.28)	−0.13 ± 0.19 (−0.76, 0.33)	−0.16 ± 0.25 (−1.00, 0.25)
1–3	−0.26 ± 0.28 (−1.13, 0.46)	−0.39 ± 1.19 (−0.88, 0.01)	−0.62 ± 0.35 (−1.48, 0.00)	−0.74 ± 0.36 (−1.70, 0.00)	−0.96 ± 0.35 (−2.15, −0.23)
3–5	−0.39 ± 0.30 (−1.36, 0.49)	−0.63 ± 0.23 (−1.16, −0.24)	−0.76 ± 0.35 (−1.84, 0.00)	−1.16 ± 0.49 (−2.84, −0.34)	−1.62 ± 0.60 (−2.86, −0.46)
5–10	−0.56 ± 0.45 (−3.44, 0.54)	−0.84 ± 0.38 (−1.85, 0.02)	−1.32 ± 0.20 (−3.98, 0.20)	−1.70 ± 0.66 (−4.20, −0.51)	−2.15 ± 0.70 (−3.76, −0.60)
10–15	−0.82 ± 0.59 (−2.87, 0.49)	−1.09 ± 0.27 (−1.65, −0.84)	−1.39 ± 0.44 (−2.58, −0.40)	−2.16 ± 0.69 (−4.10, −0.29)	−2.83 ± 0.76 (−4.58, −1.15)
15–30	−0.87 ± 0.62 (−2.53, 0.25)	−1.42 ± 0.38 (−1.88, −1.01)	−2.51 ± 0.90 (−4.73, −1.14)	−2.65 ± 0.48 (−3.49, −1.33)	−3.50 ± 0.71 (−5.06, −2.01)

**Table 3:** Univariate linear regression analysis for MBL.

Factor	Linear Equation *	*p* Value ^a^	R^2^ Linear
Smoking			
No	*y* = −0.11 − 0.00411*x*	<0.001	0.358
1–5 cig/day	*y* = −0.13 − 0.00693*x*		0.647
6–10 cig/day	*y* = −0.20 − 0.01000*x*		0.648
11–15 cig/day	*y* = −0.31 − 0.01000*x*		0.678
20 cig/day	*y* = −0.59 − 0.01000*x*		0.699
Diabetes			
No	*y* = −0.19 − 0.00796*x*	0.175	0.412
Type 1	*y* = −0.08 − 0.01000*x*		0.866
Type 2	*y* = −0.35 − 0.01000*x*		0.447
Bruxism ^b^			
No	*y* = −0.20 − 0.00773*x*	<0.001	0.388
Yes	*y* = −0.20 − 0.01000*x*		0.676
Sex			
Male	*y* = −0.19 − 0.00848*x*	<0.001	0.388
Female	*y* = −0.21 − 0.00808*x*		0.418
Age (years)			
<50	*y* = −0.27 − 0.00798*x*	<0.001	0.438
51–61	*y* = −0.21 − 0.00896*x*		0.440
>62	*y* = −0.19 − 0.00569*x*		0.212
Jaw			
Maxilla	*y* = −0.21 − 0.00828*x*	<0.001	0.348
Mandible	*y* = −0.19 − 0.00819*x*		0.456
Jaw region			
Anterior	*y* = −0.23 − 0.00816*x*	0.048	0.356
Posterior	*y* = −0.17 − 0.00827*x*		0.485
Tooth region			
Incisor	*y* = −0.23 − 0.00876*x*	0.245	0.381
Canine	*y* = −0.24 − 0.00708*x*		0.311
Premolar	*y* = −0.16 − 0.00875*x*		0.500
Molar	*y* = −0.19 − 0.00691*x*		0.467
Implant diameter			
3.00–3.50 mm	*y* = −0.23 − 0.00391*x*	0.002	0.190
3.75–4.10 mm	*y* = −0.20 − 0.00853*x*		0.429
4.30–5.00 mm	*y* = −0.49 − 0.00342*x*		0.100
Implant surface			
Turned	*y* = −0.19 − 0.00812*x*	<0.001	0.422
Modified	*y* = −0.20 − 0.00875*x*		0.361
Prosthesis type			
Single crown	*y* = −0.25 − 0.00653*x*	<0.001	0.290
FDP 2–6 units	*y* = −0.19 − 0.00828*x*		0.493
FDP 7–10 units	*y* = −0.25 − 0.00576*x*		0.363
Full-arch	*y* = −0.21 − 0.00867*x*		0.388
Overdenture	*y* = 0.08 + 0.00349*x*		0.631
Prosthesis fixation ^b^			
Cemented	*y* = −0.25 − 0.00653*x*	<0.001	0.307
Screwed	*y* = −0.21 − 0.00818*x*		0.407

* For the linear equation, “*x*” represents the number of months; ^a^ comparison of the slope of the equation (variation of MBL in mm in time) between groups; ^b^ for the cases with available information.

**Table 4:** Linear mixed-effects model for MBL.

Predictor Variables	F Statistic	*p* Value
Smoking	118.725	<0.001
Bruxism	7.216	0.007
Sex	2.118	0.103
Age	3.245	0.075
Jaw	23.786	<0.001
Jaw region	0.072	0.788
Implant diameter	4.997	0.037
Implant surface	3.576	0.068
Prosthesis type	0.105	0.705
Prosthesis fixation	6.329	0.012

## Data Availability

Restrictions apply to the availability of these data. Data were obtained from patients treated at Folktandvården Skåne AB, Malmö, Sweden, and cannot be shared, in accordance with the General Data Protection Regulation (EU) 2016/679.
